# Supplementary material for: Adrenal Gland Irradiation Causes Fatigue Accompanied by Reactive Changes in Cortisol Levels
Source: J Clin Med. 2022 Feb 24;11(5):1214. doi: 10.3390/jcm11051214 (PMC8911448; doi:10.3390/jcm11051214)
Supplement: Supplementary file 1 [file jcm-11-01214-s001.zip › jcm-1563238-supplementary.pdf]

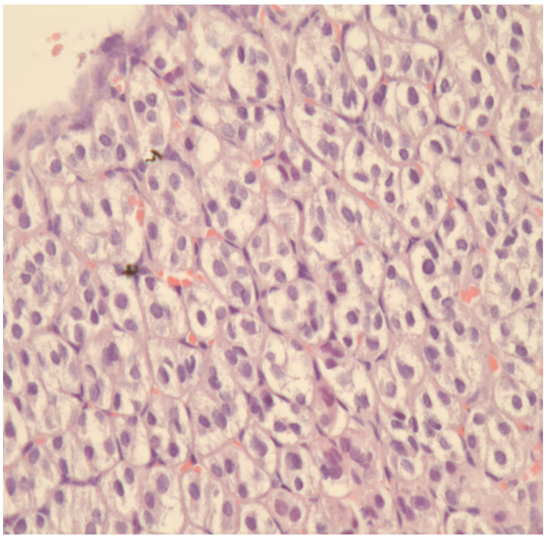
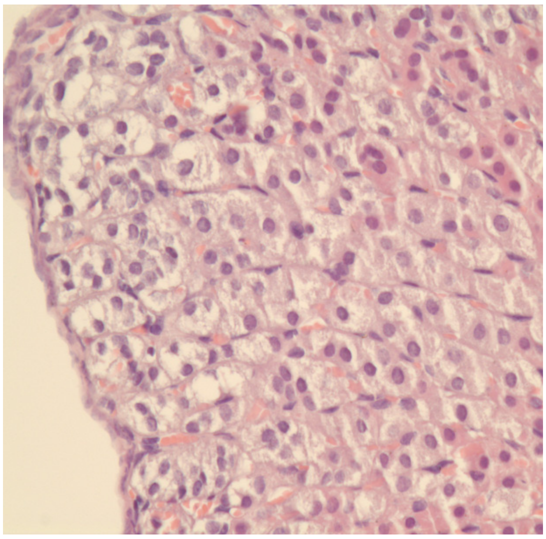

(**a**) (**b**)

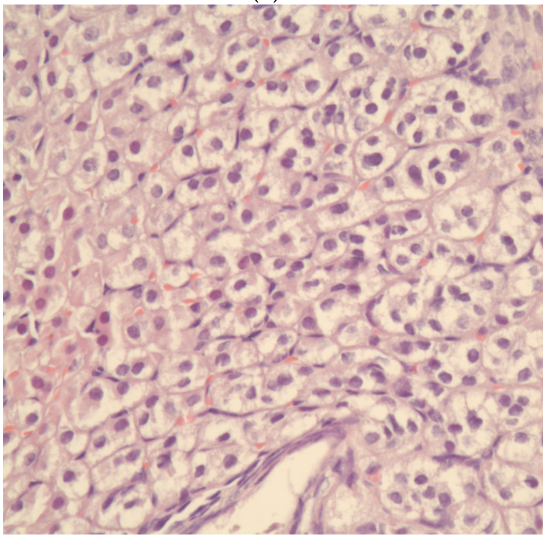
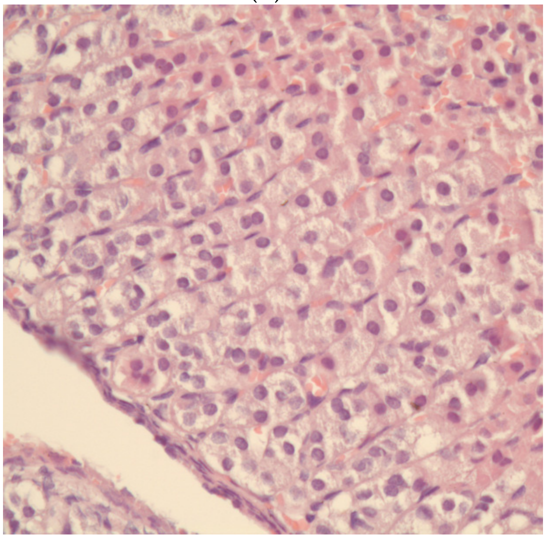

(**c**) (**d**)

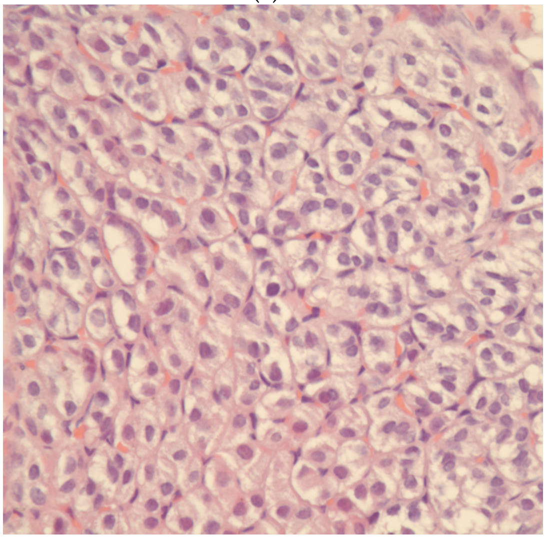
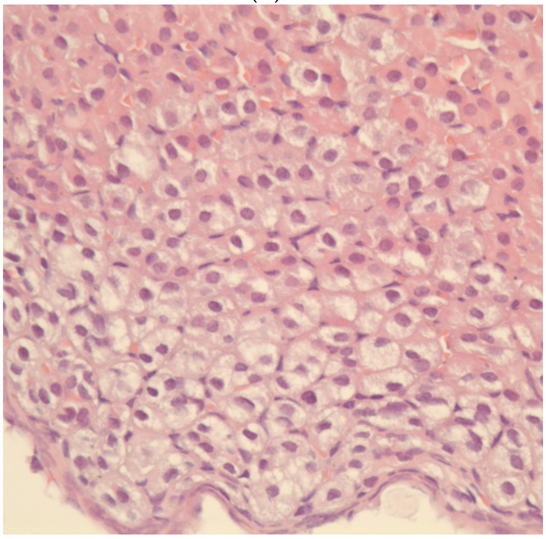

(**e**) (**f**)

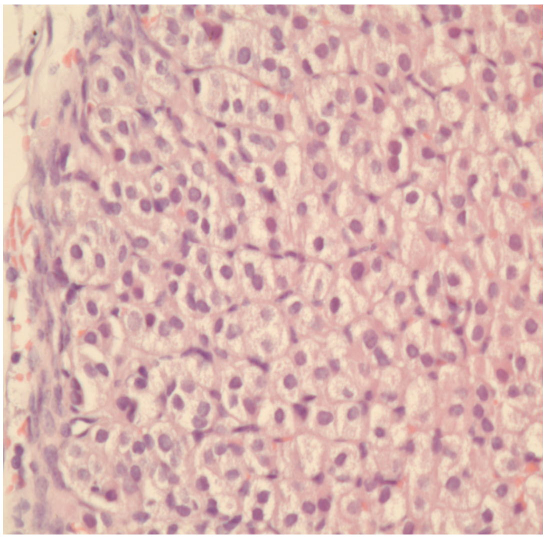

(**g**)

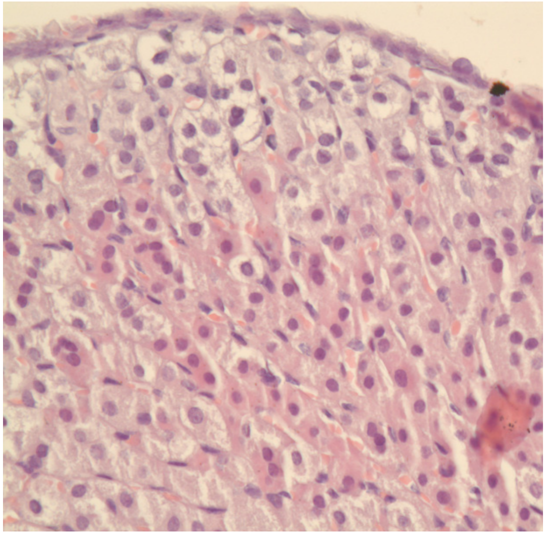

(**h**)

Supplementary Figure S1. Histopathology of adrenal cortex from each mouse in 2 Gy group by using H&E staining. Left column, unirradiated right adrenal cortex (**a**,**c**,**e**,**g**). Right column, 2 Gy irradiated left adrenal cortex (**b**,**d**,**f**,**h**). The RT-injured adrenal cortex showed moderate hypertrophy, disorganization, cellular aggregates, increased vasculogenesis, condensed chromatin in the nucleus, and cytoplasmic swelling. Abbreviations: H&E, hematoxylin and eosin; RT, radiotherapy.
